# World AIDS Day 2015

**DOI:** 10.1186/s12977-015-0228-7

**Published:** 2015-12-01

**Authors:** Andrew Lever, Mark Wainberg

**Affiliations:** Department of Medicine, University of Cambridge, Cambridge, UK; Jewish General Hospital, McGill University AIDS Centre, Montreal, QC Canada

World AIDS Day is a good time to remind the world that the infection that dominated the news headlines for so much of the 1980 and 1990s but now rarely makes even the front page of International media, is still here. It is still killing more than 3000 people per day of whom more than 500 are children. Ebola has come and almost gone but all through that outbreak AIDS continued to kill many fold more than ever Ebola did.

There is no argument that serious inroads have been made into both the death toll and medical and social havoc wreaked by HIV compared to those early days. Massive investment has led to a larger number of new drugs being discovered and brought to market over a 30 year period than has ever been the case for any human disease at any time. The number of new diagnoses of HIV infection and AIDS worldwide is declining year by year and we have learnt that this new approach to an infection which we cannot eradicate yet, that of making infected people less infectious, actually works. Pre exposure prophylaxis (PrEP), despite its detractors and the inbuilt problems with its use, drug sharing etc. is a truly viable component of reducing transmission and should be available to all high risk individuals including sex workers in the developing world. Recent results have even shown that drug resistance that had been much feared as a consequence of PrEP has been extremely infrequent and that a strategy termed PrEP on demand in which antiretroviral drugs (ARVs) are taken only in anticipation of and following sexual relations works as well as daily doses of ARVs, thereby reducing both drug side effects and costs.

Meanwhile in the wealthier world we are confronted with an almost bewildering choice of therapeutic regimes amongst which it is possible to find something that suits virtually everyone. More remarkable still is the observation that patients with HIV not only do well, they actually may have a health advantage through the intensive management of all their other medical conditions. The CHIC study in the UK now shows that a 35 year old male newly diagnosed with HIV and a CD4 count of 200, if treated appropriately and achieving a CD4 count of 350 within a year, now has a potential lifespan to age 81 which is 3 years longer than the average for an uninfected 35 year old. HIV infection teaches us many things and it has has shown that regular follow up, equivalent to comprehensive primary care, really makes a difference—quite a message for governments and health services everywhere.

World AIDS Day is thus a chance to look back and celebrate our successes in what has been arguably the most impressive medical revolution ever, where a once 100 % fatal infection can now be a chronic condition associated with a normal or even prolonged lifespan.

But, as alluded to in the opening paragraph, World AIDS Day is also a time to remind people that HIV has not gone away and in many parts of the world it is still the major cause of death, albeit often in concert with its lethal partner TB. People in the West may not fear the disease as much and the associated decline in stigma is welcome, but in rural Africa the infection rages on. Antiretroviral therapy has reached a huge proportion of this population but for many the logistics of attending a centre to gain access to treatment is still a significant barrier; the inconsistency of drug supplies—often leading to a triple regime temporarily becoming a dual or even monotherapy until supplies resume, is a continual driver for the emergence of drug resistance. Access is still linked to enrolment onto drug trials in some places and in others the fragility of ongoing funding, particularly since the withdrawal of PEPFAR and the reliance on the state and other charities, means that from 1 month to the next patients with HIV infection may not know for certain whether they will have reliable supplies of the life saving drugs which not only keep them healthy but also suppress their viral load, thereby reducing overall burden by making them less infectious.

2015 then is perhaps a watershed year. With a vaccine still as remote as ever, and even if one was shown to generate significant protection this year it would still be 5 years or more before it could be rolled out, we have to find other approaches to eliminating the virus. To that end we are now seeing the start of trials of therapies designed to eradicate HIV. These are aimed at the reservoir of latently infected cells containing virus that is untouched by current drugs. Our knowledge of the causes underlying HIV latency is growing and we are realizing the heterogeneity underlying this state. There is a long way to go before the so called ‘kick and kill’ strategies are augmented by highly specific therapies designed to selectively wake up each and every latent HIV but at least we are starting. Switching on a virus that is ‘asleep’ and which relies on normal cellular silencing processes to stay that way, while not switching on cellular genes in a dangerous and unnecessary way, is never going to be easy but every journey starts with a single step. To quote Churchill ‘This is not the end, it is not even the beginning of the end, but it may be the end of the beginning’.

Mark Wainberg.

Andrew Lever.

